# Electrically Responsive Shape Memory Composites Using Silver Plated Chopped Carbon Fiber

**DOI:** 10.3389/fchem.2020.00322

**Published:** 2020-05-08

**Authors:** Yongkun Wang, Zhenhong Chen, Jiahao Niu, Yang Shi, Jiangpeng Zhao, Junjie Ye, Wenchao Tian

**Affiliations:** ^1^Key Laboratory of Ministry of Education for Electronic Equipment Structure Design, Xidian University, Xi'an, China; ^2^College of Textile and Garment, Hebei University of Science and Technology, Shijiazhuang, China; ^3^Xi'an Research Institute of China Coal Technology, Engineering Group Corp, Xi'an, China

**Keywords:** silver plated chopped carbon fiber, hydro-epoxy, thermal conductivity, electrical conductivity, electroactive shape memory behavior

## Abstract

High electrical and thermal conductivity are beneficial to the shape recovery performance of electroactive shape memory polymer composites. In this work, the chopped carbon fiber (CCF) was processed into silver plated chopped carbon fiber (Ag/CCF), and the Ag/CCF was filled into hydrogenated bisphenol A epoxy (H-EP) resin to fabricate the electro-induced shape memory polymer composites. The Ag/CCF/H-EP composites show good electrical and thermal conductivity compared to the CCF/H-EP composites. When the content of Ag/CCF reaches 1.8 wt%, the e Ag/CCF/H-EP composites reach the threshold of thermal conductivity, electrical conductivity and percolation. The thermal conductivity of H-EP composite with 5.4 wt% Ag/CCF is 2.33 W/(m·K), which is 2.6 times and 12 times of that of CCF/H-EP composite and H-EP matrix, respectively. When the content of Ag/CCF reaches 7.2 wt%, the volume resistivity of Ag/CCF/H-EP composites decrease from 1.69 × 10^16^ Ω·to 9.51 × 10^3^ Ω cm, and surface resistivity from 6.91 × 10^15^ Ω to 6.19 × 10^2^ Ω, respectively. And the Ag/CCF/H-EP composites show good mechanical properties and dynamic thermomechanical properties. When the content of Ag/CCF is more than 1.8 wt%, the Ag/CCF/H-EP composites exhibit excellent electroactive shape memory performance, and the shape recovery rate of the composites is more than 92%.

## Introduction

Shape memory polymers (SMPs) are a class of intelligent response materials that have developed rapidly in recent years. They are able to temporarily transform their shapes and return to permanent shapes with appropriate stimulation, such as temperature, electrical, magnetic fields, light, moisture, solvent, pH, etc. (Lu et al., 2015b; Liu et al., 2017; Wang et al., 2017a; Zhang and Serpe, 2017; Zheng and Xie, 2017; Mittal et al., 2018; Yao et al., 2018a). This memory property provides huge opportunities for SMP applications in areas such as smart biomedical materials, smart textiles, sensors and drives, information carriers and aerospace (Lu et al., 2014a; Leng et al., 2015; Yao et al., 2015; Boyraz et al., 2018; Liu et al., 2018; Persson et al., 2018; Du et al., 2019; Kim et al., 2019). Therefore, many researchers have been devoted to the synthesis of new SMPs and investigating the shape memory effects (SME) of new SMPs, or endowing common polymers with SME to expand their application fields (Jin et al., 2018; Li X. et al., 2019).

Compared with shape memory alloys and shape memory ceramics, SMPs have more useful properties, such as simple processing, low density, low cost, and a wide range of shape recovery temperatures. Therefore, SMPs have attracted more and more research interest (Wang Y. et al., 2016; Kausar, 2017; Lan et al., 2018; Yao et al., 2018b; Huang et al., 2019). Generally, SMPs are divided into thermoplastic SMPs and thermoset SMPs. Compared with thermoplastic SMPs, thermoset SMPs have higher stiffness and dimensional stability, and have better environmental durability. Therefore, thermoset SMPs have become common matrix materials in structural composites due to their high stiffness and high recovery force (Zheng et al., 2016). It is worth mentioning that shape memory epoxy is a widely used thermosetting plastic with many excellent properties: high mechanical strength and thermal stability, good resistance to acid and alkali corrosion, and good formability (Hu et al., 2012). Moreover, the shape recovery temperature of shape memory epoxy can be adjusted within a certain range, and shape memory epoxy with different *T*_g_ can be prepared by compounding.

Although thermoset SMPs have outstanding performance, their low mechanical strength and shape recovery stress still limit their application. In order to solve this problem, researchers filled high modulus inorganic or organic fillers into SMPs to improve their mechanical properties. It is worth mentioning that carbon materials are the most widely used fillers, including carbon black, carbon fiber, carbon nanotubes, carbon nanopapers, graphene, and their combined nanofillers (Lu et al., 2015a, 2016; Yao et al., 2016; Wang et al., 2017b, 2018, 2020; Feng et al., 2020a). Li et al. point out that carbon fillers have found great applications in polymer materials and produced a series of fascinating multifunctional composite materials. Among the various material properties of composite materials that have been reinforced with carbonaceous material, electrical conductivity and mechanical properties are two key factors for evaluating the effectiveness of fillers in polymer matrices (Li Y. et al., 2019). In addition, carbon fibers are known to have excellent electrical conductivity and mechanical property. Therefore, doping the carbon fibers into SMPs does not only improve the mechanical properties of SMPs, but also improves its conductivity. SMPs with good conductivity can be prepared into electroactive SMPs. Electrical response is a very convenient stimulus, especially in applications where direct heating is inconvenient or difficult to achieve, so electrical response SMPs have been extensively studied (Wang K. et al., 2016; Feng et al., 2020b).

However, most of the literature only focuses on the electrical and mechanical properties of the electrically induced SMPs. Essentially, the shape recovery of electrical response SMPs is stimulated by the heat generated by electricity. A certain voltage is applied to the electrical response SMPs. Due to the Joule heating effect, electrical energy is converted into thermal energy, causing the temperature to exceed its shape transition temperature, thereby activating SMPs shape recovery (Sun et al., 2019). This shows that the thermal conductivity of electrically induced SMPs is also a very important factor. This is because good thermal conductivity helps electrically induced SMPs to reach shape memory transition temperature in a short time, which is conducive to improving the recovery speed of electrically induced SMPs. In this work, we attempt to electroplate a layer of silver on the surface of chopped carbon fiber (CCF) to prepare silver plated chopped carbon fiber (Ag/CCF), in order to improve the conductivity and thermal conductivity of CCF. Then, Ag/CCF was filled into the shape memory hydro-epoxy (H-EP) resin to fabricate electroactive shape memory composites. And the effects of Ag/CCF on the mechanical properties, electrical properties, thermal properties, and thermo-mechanical properties of Ag/CCF/H-EP composites were studied.

## Experimental

### Materials

The epoxy resin (AL3040, epoxy value 0.43 eq/100 g) was purchased from Complex high tech materials Co., Ltd. (Shanghai, China). The curing agent methyl tetrahydro-phthalic anhydride (MeTHPA, molecular weight 166.181 Da) was provided by Wenzhou Qingming Chemical Co., Ltd (Wenzhou, China). The accelerant agent 2,4,6-tris (dimethylaminomethyl) phenol (DMP-30, molecular weigh 265.4 Da) was purchased from Guangzhou Weilina Chemical Co., Ltd (Guangzhou, China). CCF, conductivity is about 0.001 Ω·cm, length is 200 μm, diameter is 4–6 μm, was purchased from Toray Co., Ltd. (Japan). The sodium hydroxide (NaOH), stannous chloride (SnCl_2_), silver nitrate (AgNO_3_), palladium dichloride (PdCl_2_) and ammonia (NH_3_·H_2_O) were provided by Tianjin Chemical Reagent Factory (Tianjin, China).

### Fabrication of Ag/CCF

**Figure 1:**
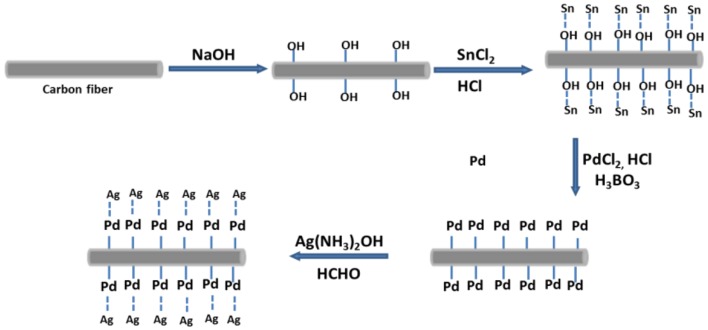
Preparation process of the Ag/CCF.

Firstly, the CCF was placed in a muffle furnace and calcined at 300°C for 10 min to remove the organic impurities on the surface, then the adhesion was removed by ultrasonic in ethanol solution for 30 min, and then cleaned and dried. Secondly, the surface of CCF was roughened by ultrasonic dispersion in NaOH solution for 1 h, and then the surface of CCF was sensitized by immersion in the mixed solution of SnCl_2_ and HCl for 1 h at room temperature. The Sn_2_(OH)_3_Cl generated in the solution is adsorbed on the CCF surface, filtered and washed, and then activated with the mixed solution PdCl_2_, H_3_BO, and HCl for redox reaction. Finally, AgNO_3_ was dissolved in a deionized aqueous solution, and ammonia water was added dropwise to prepare a silver ammonia solution. The surface-treated CCF was dispersed in the prepared silver ammonia solution, and HCHO solution was slowly added, with continuous stirring. After reaction at room temperature for 30 min, the CCF was filtered, washed and dried, and finally Ag/CCF was obtained. The above steps are shown in Figure 1.

### Preparation of Electrically Responsive Shape Memory Composites

**Scheme 1:**
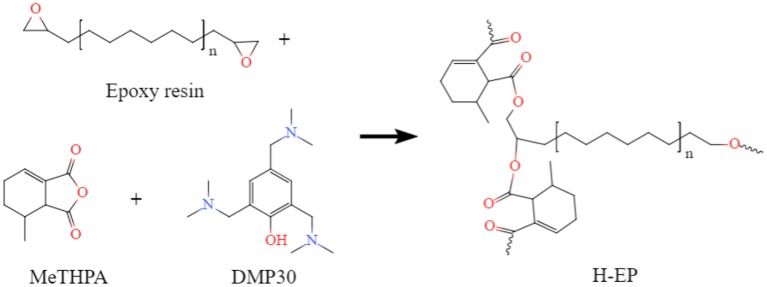
Cross-linked of hydro-epoxy.

Ag/CCF was added to 200 mL of acetone, and then the mixture was ultrasonic treated at 50°C for 1 h to disperse the Ag/CCF. After ultrasonic treatment, Ag/CCF was added to epoxy resin stored at 60°C. At this temperature, the epoxy resin has good fluidity, is easy to mix with the filled Ag/CCF, and prevents the treated Ag/CCF from reuniting in the epoxy resin. The mixture of Ag/CCF/epoxy resin was stirred with a mechanical stirrer, and then ultrasonic treatment was carried out for 2 h. After that, the curing agent MeTHPA and accelerator DMP-30 (the weight ratio is epoxy resin: MeTHPA: DMP-30 = 100: 80: 0.8) were successively added to the mixture. The addition amount of Ag/CCF per 100 g epoxy resin is 0, 1.8, 3.6, 5.4, and 7.2 g respectively. Finally, the mixture of Ag/CCF/epoxy/MeTHPA/DMP-30 was poured into the glass mold for curing. A three-step curing procedure was used: 80°C/1 h + 110°C/3 h + 150°C/2 h. The crosslinking process of H-EP is shown in Scheme 1. The sample of neat epoxy resin and the epoxy composites with CCF were also prepared for comparison by the same process.

### Experimental Methods

#### Flexural Testing

According to the ASTM D7264 test method, a bending test was performed using CMT5105 (Shenzhen SANS Testing Machine Co., Ltd, Shenzhen, China) with a load of 10 kN and a speed of 2 mm/min. Measurements were made on injection spline specimens measuring 100 × 10 × 2 mm^3^, and the average of at least 3 individual values was used.

#### Impact Testing

The impact test was performed using a Charpy impact tester BC-50 (China Shenzhen Sansi Co., Ltd.) in accordance with ASTM D7136. Measurements were made at an impact speed of 2.9 m/s, and an average of five individual values was used.

#### Thermal Conductivity

The samples were cut into 20 × 20 × 2 mm^3^ and tested with a thermal constant analyzer (Hot-Disk, TPS2500S, Switzerland).

#### Electrical Conductivity Tests

According to the GB/T 1410-2006 test method, the resistivity and surface resistivity of Ag/CCF/H-EP composites were measured using a four-probe metal/semiconductor resistivity meter (HAD-SB 100A/2, Beijing hengaode Instrument Co., Ltd., Beijing, China).

#### SEM

The morphology of Ag/CCF was analyzed by SEM (JSM-6390, HITACHI, Japan). The Ag/CCF/H-EP composites samples were frozen in liquid nitrogen, and then brittle fracture occurred. SEM (JSM-6390, HITACHI, Japan) observation of the fractured surface was completed using the sputtering gold plating method.

#### Energy Conversion Dispersive Spectrometer (EDS)

The composition of Ag/CCF was characterized by an EDS analyzer (JED-2200 F, Japan).

#### X-Ray Diffraction Analysis (XRD)

The grain size, cell parameters and crystal structure of Ag layer on Ag/CCF surface were measured by X-ray diffractometer (PANalytical X'Pert Pro, Netherlands). The tube voltage is 30 kV, the tube current is 25 mA, the X-ray source is Cu target (λ = 0.15406 nm), the scanning range is 10–80° and the scanning step is 4°/ min.

#### Dynamic Mechanical Analysis (DMA)

DMA tests use a DMA Q800 analyzer (TA Instrument, USA) to perform a three-point bending test at a heating rate of 5°C/min and a load frequency of 1 Hz. A rectangular sample with a size of 60 × 10 × 2 mm^3^ was used for measurements in the range of 25–200°C.

#### Electroactive Shape Memory Property Testing

**Figure 2:**
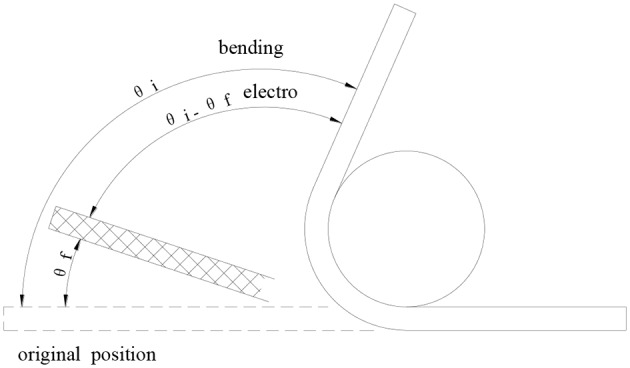
The electrical response shape memory model.

The rectangular samples (100 × 10 × 2 mm^3^) were used to test the electrical response shape memory performance of the Ag/CCF/H-EP composites. The shape memory model is shown in Figure 2. The electrical response shape memory test was performed using the following steps: (i) heating the samples to a target temperature T_g_ in the oven and holding them for 10 min; (ii) bending the samples into “U” shape at a bending rate of 30° s-1 around a center axis with a diameter of 10 mm. The “U” shaped samples were then quickly removed from the oven and immersed in a cold-water bath with a constant external force. At this time, the sample will have a small elastic recovery, the deformation angle becomes θ_*fix*_, and the shape fixation rate is defined as θ_*fix*_/θ_*i*_;(iii) applying different voltages to observe the shape recovery process of the samples. When the samples no longer change, record the recovery time. The deformation recovery speed is defined as (θ_*i*_ − θ_*f*_)/*t*, and the deformation recovery rate is defined as (θ_*i*_ − θ_*f*_)/θ_*i*_ × 100%.

## Results and Discussion

### Structures of Ag/CCF

**Figure 3:**
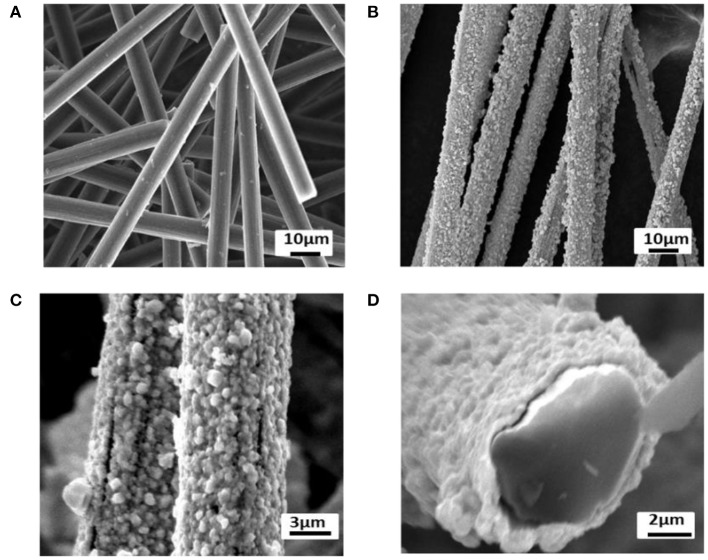
SEM of **(A)** CCF, **(B,C)** Ag/CCF, and **(D)** cross sections Ag/CCF.

During the preparation of Ag/CCF, the surface morphology of CCF is shown in Figure 3. Figure 3A shows that the average length of CCF is about 200 μm and the average diameter is about 5 μm. Figures 3B,C show that the Ag layer is evenly and tightly covered on the CCF surface, without plating vacancy. From Ag/CCF cross section in Figure 3D, it can be estimated that the thickness of the Ag layer is about 450 nm.

**Figure 4:**
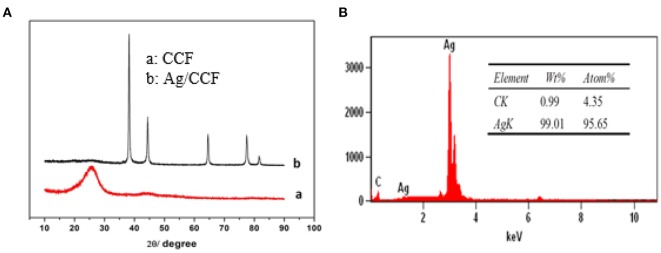
**(A)** XRD of CCF and Ag/CCF, **(B)** EDS of Ag/CCF.

The crystal structure of CCF and Ag/CCF were analyzed by XRD, and the results are shown in Figure 4A. The five peaks at 2θ = 38.22, 44.40, 64.56, 77.44, and 81.60° correspond to (111), (200), (220), (311), and (222) crystal faces of Ag, respectively, but the oxidation state of Ag has not been detected. The diffraction peak of carbon appears at 2θ = 25.56°, which is not observed in the diffraction characteristic peak of silver plated chopped carbon fiber, further indicating that the surface of CCF has been completely coated with Ag. According to the Debye-Scherrer equation, the thickness of the Ag layer is calculated to be 460 nm, which is consistent with the value measured by SEM.

In order to further explain the coating of CCF surface by Ag, EDS was used to analyze the surface elements of Ag/CCF. Figure 4B shows the EDS spectrum of Ag/CCF, and the table shows the surface components and their contents of Ag/CCF. There are mainly Ag and C elements in Ag/CCF, of which the mass content of metal Ag is as high as 99.01% and the atomic coverage rate is 95.65%. It can be seen that the surface of Ag/CCF is almost coated with Ag, and the detection of oxygen-free elements fully shows that there is no oxidation of silver in the preparation process of silver plating of CCF.

### Characteristics of the Ag/CCF/H-EP Composites

#### The SEM of Ag/CCF/H-EP Composites

**Figure 5:**
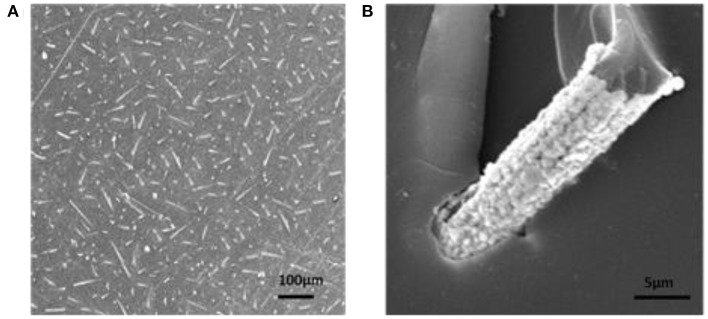
**(A)** Cross-sectional of Ag/CCF/H-EP composite, **(B)** Ag/CCF embedded into H-EP matrix.

The cross section of Ag/CCF/H-EP composite with 3.6% Ag/CCF filling is shown in Figure 5A. From the Figure 5A, the bright part is Ag/CCF, and the black part is H-EP matrix. Ag/CCF is uniformly distributed in H-EP matrix without agglomeration. The disordered distribution of the homogeneity of Ag/CCF is beneficial to the uniform heat transfer in the direction of heat flow. Figure 5B is single Ag/CCF embedded into EP matrix.

#### Thermal Conductivity of Ag/CCF/H-EP Composites

**Figure 6:**
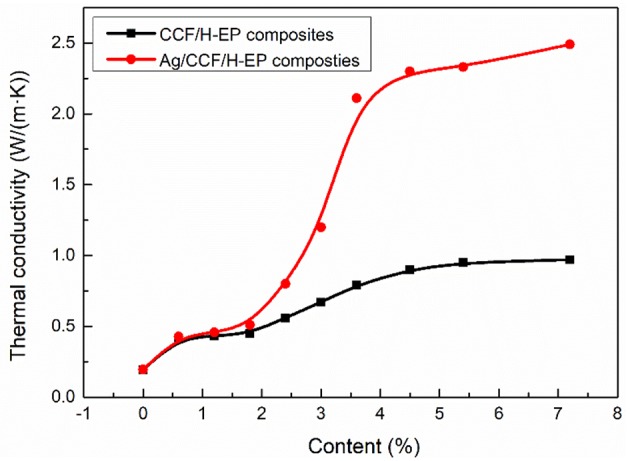
Thermal conductivity of Ag/CCF/H-EP composites and CCF/H-EP composites.

The thermal conductivity of CCF/H-EP and Ag/CCF/H-EP composite filled with pure CCF and Ag/CCF, respectively, is shown in Figure 6. With the increase of fillers, CCF/H-EP and Ag/CCF/H-EP have the same increasing trend of thermal conductivity. It can be seen from Figure 6 that when the filler content is <1.8 wt%, the thermal conductivity of the composite increases slowly, and when it reaches 1.8 wt%, the thermal conductivity of the composite increases sharply. Therefore, the threshold of thermal conductivity and percolation of the two composites is 1.8 wt%.When the filler is <1.8 wt%, the fillers are isolated by the thick layer of resin matrix, and there is a large interface thermal resistance between fillers. When the filler content exceeds 1.8 wt%, the fillers contact each other, or are isolated by a thin resin matrix. Therefore, a three-dimensional heat conduction path can be formed between the fillers in the resin matrix, thereby rapidly increasing the thermal conductivity. The thermally conductive filler has higher thermal conductivity than the resin matrix, and heat flow propagates between the fillers. When the thermally conductive filler is >5.4 wt%, the thermal conductivity increases slowly. At this time, an effective thermal conduction network within the resin matrix has been formed, and the thermal conduction path tends to saturation.

Since the resin matrix does not have freely moving electrons, the heat flow is transmitted through the phonons. The heat flow is transmitted inside the H-EP with low crystallinity, and the phonon scattering causes the thermal conductivity to be as low as 0.21 W/(m·K). Ag is a good conducting material. There are a lot of movable electrons in the atom, which can also be used as a good conductor of heat. The Ag layer on the surface of carbon fiber can improve the thermal conductivity of CCF and reduce the contact thermal resistance. It can be seen from Figure 6 that the thermal conductivity of /H-EP composite containing 5.4 wt% Ag/CCF is 2.33 W/(m·K), which are the thermal conductivity of the CCF/H-EP composites and H-EP matrix 2.6 times and 12 times, respectively. When the Ag/CCF content increased to 7.2 wt%, the thermal conductivity of the composite increased to 2.50 W/(m·K). In the literature, the thermal conductivity of graphene/EP composite is only 1.53 W/(m·K) when filled with 10 wt% graphene (Song et al., 2013), and that of MWCNT/polyurethane composite is only 0.47 W/ (m · K) when filled with 3.0 wt% filler (Cai and Song, 2008).

#### Electrical Conductivity of Ag/CCF/H-EP Composites

**Figure 7:**
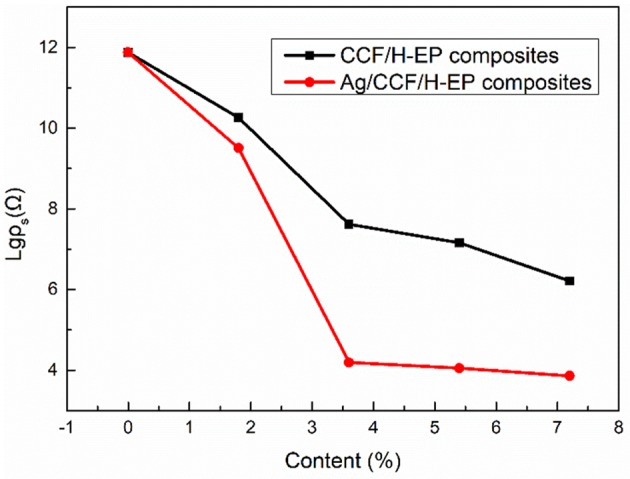
Effect of different filling amount on the surface resistivity (ρ_s_) of composites.

**Figure 8:**
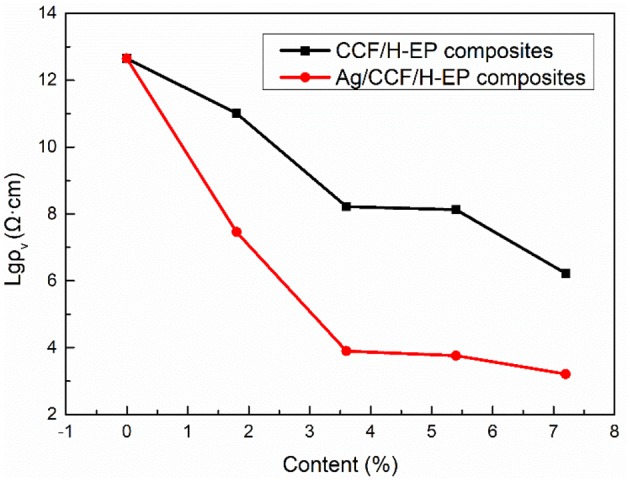
Effect of different filling amount on the volume resistivity (ρ_v_) of composites.

Both Ag/CCF and CCF have conductivity, which can improve the conductivity of the composite. As shown in Figures 7, 8, as the filler content increases, the resistivity of Ag/CCF/H-EP composite and CCF/H-EP composite decrease significantly. However, since Ag/CCF has better conductivity than CCF, the volume resistivity (ρ_V_) of Ag/CCF/H-EP composite is lower than that of CCF/H-EP composite at the same amount. When the filler content reaches 7.2 wt%, the volume resistivity of the Ag/CCF/H-EP composites decrease from 1.69 × 10^16^ Ω to 9.51 × 10^3^ Ω·cm, and the surface resistivity (ρ_S_) decreases from 6.91 × 10^15^ Ω to and 6.19 × 10^2^ Ω.

#### Mechanical Properties of Ag/CCF/H-EP Composites

Although SMPs have been applied in some fields, they still have the limitation of low recovery stress. Therefore, it is necessary to test the mechanical properties of composites. The impact strength and flexural strength of the Ag/CCF/H-EP composites were tested at room temperature (25°C). Since the conductivity and thermal conductivity of Ag/CCF/H-EP composites are better than CCF/H-EP composites, the mechanical analysis is only performed on Ag/CCF/H-EP composites.

**Figure 9:**
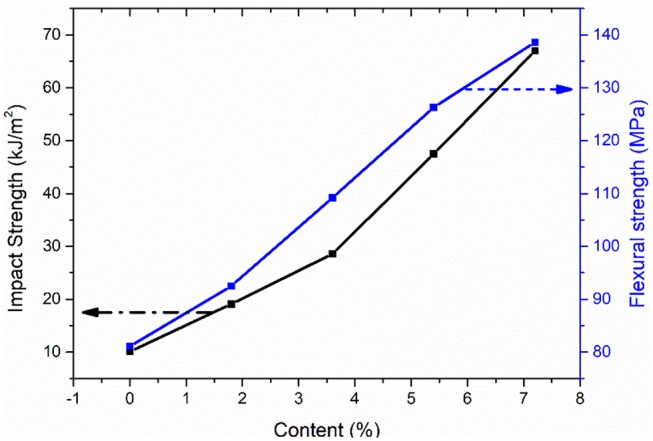
Impact strength and flexible strength of Ag/CCF/H-EP composites.

The impact properties can reflect the ability of composites to resist crack growth and brittle fracture (Ye et al., 2019a,b). Therefore, it is necessary to test the impact performance of composites. The effect of Ag/CCF content on the impact strength and flexural strength of Ag/CCF/H-EP composite is shown in Figure 9. It is worth noting that the addition of Ag / CCF significantly improved the impact strength and flexural strength of the composites. The flexural strength and impact strength of composite increase with increasing Ag/CCF content. The impact strength and flexural strength of pure H-EP matrix are 10.2kJ/m^2^ and 81.0MPa, respectively. When the Ag/CCF content is increased to 7.2 wt%, the flexural strength and impact strength of the material are 138.5 MPa and 67.1 KJ/m^2^, respectively, which are about 563% and 70.9% improved compared with that of pure resin matrix. Ag/CCF filler can improve the mechanical strength of the composites effectively, the main reason is that the resin matrix will produce micro cracks in the composite when it is stressed. The Ag/CCF fillers dispersed in the resin matrix can effectively prevent the growth of micro cracks and absorb the expansion energy. Thus, the prepared Ag/CCF/H-EP composites have great application prospects in intelligent devices.

#### DMA of Ag/CCF/H-EP Composites

**Figure 10:**
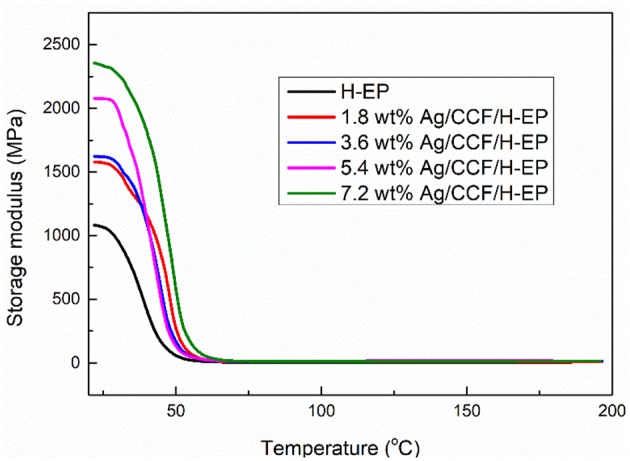
Storage modulus curves of Ag/CCF/H-EP composites.

Below the shape memory transition temperature, the storage modulus of the composite is related to the shape fixation rate, and above the shape memory transition temperature, the storage modulus of the composite is related to the shape recovery ratio (Liu et al., 2017). The storage modulus of the Ag/CCF/H-EP composites are shown in Figure 10. It can be found in Figure 10 that the storage modulus increases with increasing Ag/CCF content. Generally, the storage modulus of composites with excellent shape memory properties is more than 2–3 orders of magnitude lower than the shape transition temperature than above the shape memory transition temperature (Wang et al., 2017b). Figure 10 shows that the storage modulus of Ag/CCF/H-EP composites below the shape memory transition temperature is approximately three orders of magnitude larger than that of Ag/CCF/H-EP composites that are above the shape memory transition temperature. This shows that Ag/CCF/H-EP composite is an ideal shape memory composite.

**Figure 11:**
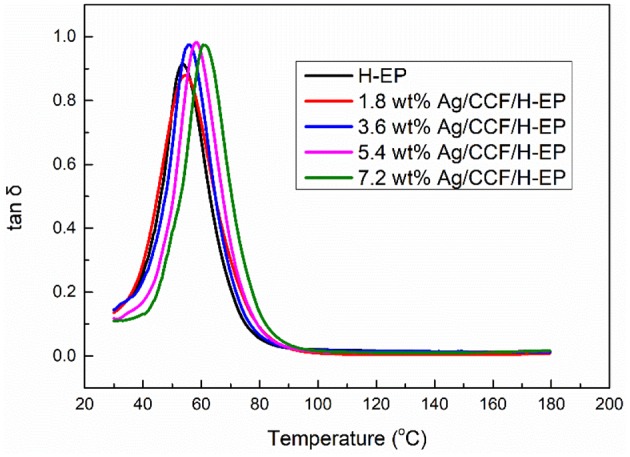
Tan δ curves of Ag/CCF/H-EP composites.

Shape memory transition temperature is a very important parameter for shape memory performance. For thermoset SMPs, the general glass transition temperature (T_g_) is the shape memory transition temperature. In this work, the T_g_s of the Ag/CCF/H-EP composites are the peaks of tan δ curves, as shown in Figure 11. From Figure 11, it can be found that with the increase of the Ag/CCF content, the T_g_s of the Ag/CCF/H-EP composites increase slightly, and the T_g_ of H-EP is 53.5°C. When the Ag/CCF content is 1.8, 3.6, 5.4, and 7.2wt%, the *T*_g_ of the Ag/CCF/H-EP composite is 54, 56, 58.5, and 61.5°C, respectively. This is due to the existence of certain hydrogen bonds between the Ag/CCF and H-EP matrix dispersed in the resin matrix, which restricts the movement of the polymer segment and causes the T_g_s of the composites to increase.

#### Electroactive Shape Recovery Behavior

**Figure 12:**
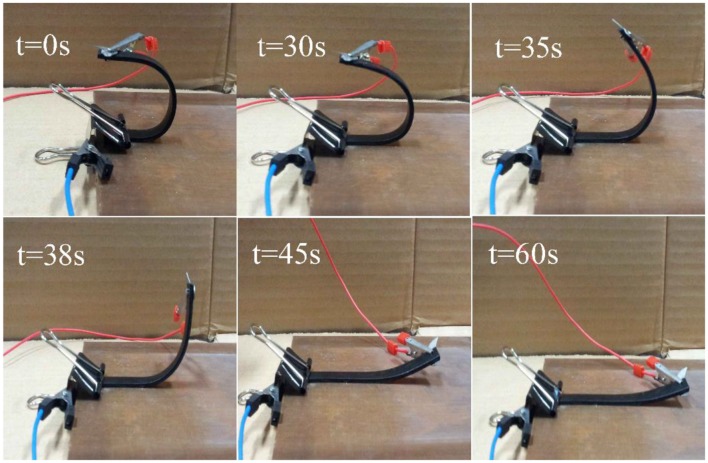
Shape memory of the Ag/CCF/H-EP composite with 5.4 wt.% Ag/CCF activated by electricity under 60 V.

The temporary *U*-shape was stimulated with a voltage of 60 V, and the electrical response shape memory characteristics of H-EP composites containing 5.4 wt% Ag/CCF were studied, as shown in Figure 12. The Figure 12 shows the shape recovery process of 5.4 wt% Ag/CCF/H-EP composite at 0, 30, 35, 38, 45, and 60 s, respectively. The results show that electroactive property of Ag/CCF/H-EP composites can be accomplished with *R*_r_ of 95.3%.

**Figure 13:**
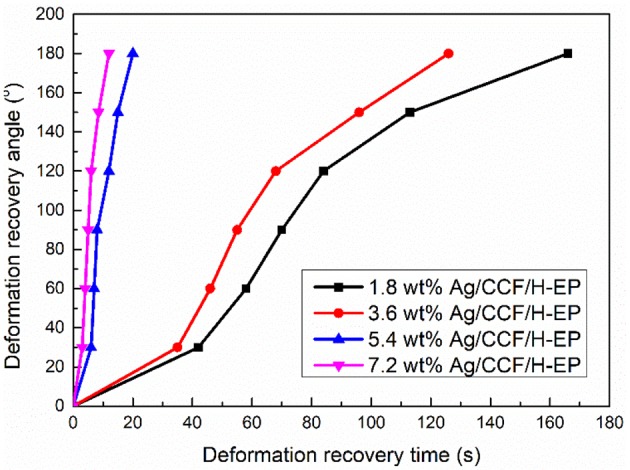
Relationship between deformation recovery angle and deformation recovery time of Ag/CCF/H-EP composites under 120 V.

To analyze the effect of Ag/CCF content on the electroactive shape memory properties of the composites, the relationship between the deformation recovery time and the deformation recovery angle of the composite at 120 V were tested. The slope of the deformation recovery angle and the deformation recovery time is the deformation recovery speed, and the relationship curve is shown in Figure 13. It can be seen from Figure 13 that with the increase of the Ag/CCF content, the deformation recovery time of the composites is significantly shortened. Meanwhile, from the curve of Ag/CCF content of 1.8 and 3.6 wt%, it can be seen that when the recovery angle is 30–120°, the deformation recovery speed of composites is fast, while at 0–30 and 150–180°, the deformation recovery speed of composites is slow. This is because in the initial stage of shape recovery, the composites need to generate heating by electricity and when the temperature reaches the T_g_s, the stress of the composites freezing can be gradually released, thereby triggering the shape recovery. Then, the rapid release of the frozen stress of the composites cause rapid recovery of the deformation. In the final stage of the shape recovery, the frozen stress is gradually released, resulting in a slower rate of deformation recovery.

**Figure 14:**
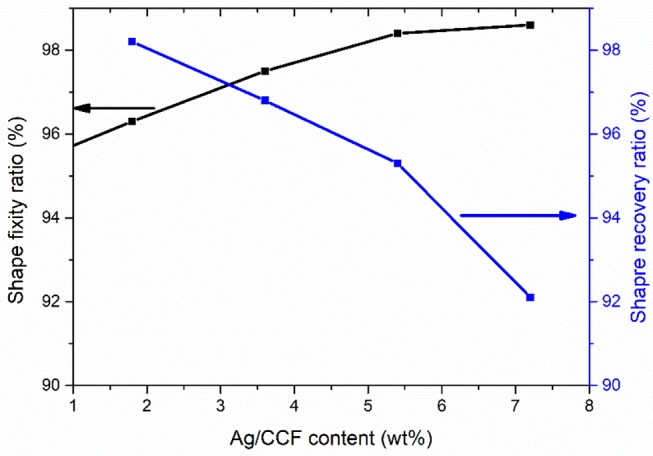
Shape fixity ratio and shape recovery ratio of the Ag/CCF/H-EP composites.

Because the electroactive shape memory performance is excited by the conversion of electrical energy into Joule heat, the generation of Joule heat depends on the distribution state and content of the conductive filler in the SMP matrix (Liu et al., 2009; Meng and Li, 2013). Therefore, the formation of a conductive network in a composite is a prerequisite for endowing the Ag/CCF/H-EP composites with electroactive shape memory properties. In Ag/CCF/H-EP composites, Ag/CCF is wrapped by an insulating H-EP matrix, which hinders the transmission of electricity and thermal to a certain extent. Therefore, when the content of Ag/CCF in the Ag/CCF/H-EP composite is low, the Ag/CCF cannot form a good conductive network, which results in the Ag/CCF/H-EP composite having no electroactive shape memory effect. As shown in Figure 14, when the Ag/CCF content is more than 1.8 wt%, the composite sample can almost return to its original shape at a certain voltage. However, as the content of Ag/CCF increases, the shape recovery rate of the composite gradually decreases. This is because Ag/CCF does not have SME, and the addition of Ag/CCF will hinder the molecular chain movement of the H-EP matrix. It worth noting that the shape recovery rate of all samples was above 92%.

Shape fixity ratio is an important parameter that affects shape memory behavior. It represents the ability of SMPs to keep its shape during the secondary molding. And the value of shape fixity ratio directly affects the storage and safekeeping of the secondary molding products in use. The shape fixity ratio of composites is shown in Figure 14, it can be found that as the content of Ag/CCF increases, the shape fixation rate of the composites increases significantly, and the shape fixation rates of all composites are above 96%. This is because Ag/CCF can suppress plastic deformation of the composite. From the literature, the shape recovery ratio of reduced graphene oxide/carbon fibers/ epoxy composites is 95% (Lu et al., 2014b), and the shape fixation rate of carbon nanofiber/epoxy shape memory composite is about 98% (Dong et al., 2014), which demonstrates that Ag/CCF/H-EP composites have excellent shape memory effects.

The effect of applied voltage on the shape memory performance of composite materials is shown in Table 1. As can be seen from Table 1, as the applied voltage increases, the deformation recovery time is greatly shortened. When the applied voltage is 160 V, the 7.2 wt% Ag/CCF/H-EP composite can achieve deformation recovery in 9 s. However, when the voltage exceeds 160 V, the sample is liable to cause burning.

**Table 1:** Recovery time of the composites under different voltage.

**Samples**	**60 V**	**80 V**	**100 V**	**120 V**	**140 V**	**160 V**
1.8 wt% Ag/CCF/H-EP	—	—	—	166 s	135 s	102 s
3.6 wt% Ag/CCF/H-EP	—	—	158 s	126 s	98 s	87 s
5.4 wt% Ag/CCF/H-EP	60 s	51 s	38 s	20 s	18 s	17 s
7.2 wt% Ag/CCF/H-EP	56 s	45 s	29 s	12 s	10 s	9 s

## Conclusion

In this study, a novel electrically responsive shape memory polymer composite constituted by Ag/CCF and H-EP was successfully developed. The Ag/CCF was synthesized by an electroless plating method, the silver coating appears homogenously deposited on the CCF. When the composite is doped with 7.2 wt% Ag/CCF, the thermal conductivity of the composite can reach 2.50 W/(m·K), the surface resistivity can reach 6.19 × 10^2^ Ω·cm, and the volume resistivity can reach 9.51 × 10^3^ Ω. The mechanical properties, storage modulus and T_g_s of the composites all increased with the increment of Ag/CCF content. When the Ag/CCF content exceeds 1.8 wt%, the composites have excellent electroactive shape memory effect. The shape recovery speed of the composite accelerated with the increase of the applied voltage and the Ag/CCF content, the shape recovery ratio exceeded 92% and the shape fixation rate was more than 95%.

## Data Availability Statement

All datasets generated for this study are included in the article/supplementary material.

## Author Contributions

Experiments conceived and designed and the paper written by YW. Experiments performed by YW, ZC, and JN. YS, JZ, JY, and WT analyzed the data.

## Conflict of Interest

The authors declare that the research was conducted in the absence of any commercial or financial relationships that could be construed as a potential conflict of interest.
